# Unravelling the role of the group 6 soluble di‐iron monooxygenase (SDIMO) SmoABCD in alkane metabolism and chlorinated alkane degradation

**DOI:** 10.1111/1751-7915.14453

**Published:** 2024-04-29

**Authors:** Eleonora Ferrari, Giulio Di Benedetto, Andrea Firrincieli, Alessandro Presentato, Dario Frascari, Martina Cappelletti

**Affiliations:** ^1^ Department of Pharmacy and Biotechnology (FaBit) University of Bologna Bologna Italy; ^2^ Department for Innovation in Biological, Agro‐Food and Forest Systems University of Tuscia Viterbo Italy; ^3^ Department of Biological, Chemical and Pharmaceutical Sciences and Technologies (STEBICEF) University of Palermo Palermo Italy; ^4^ Department of Civil, Chemical, Environmental and Materials Engineering (DICAM) University of Bologna Bologna Italy

## Abstract

Soluble di‐iron monooxygenases (SDIMOs) are multi‐component enzymes catalysing the oxidation of various substrates. These enzymes are characterized by high sequence and functional diversity that is still not well understood despite their key role in biotechnological processes including contaminant biodegradation. In this study, we analysed a mutant of *Rhodoccocus aetherivorans* BCP1 (BCP1‐2.10) characterized by a transposon insertion in the gene *smoA* encoding the alpha subunit of the plasmid‐located SDIMO SmoABCD. The mutant BCP1‐2.10 showed a reduced capacity to grow on propane, lost the ability to grow on butane, pentane and *n‐*hexane and was heavily impaired in the capacity to degrade chloroform and trichloroethane. The expression of the additional SDIMO *prmABCD* in BCP1‐2.10 probably allowed the mutant to partially grow on propane and to degrade it, to some extent, together with the other short‐chain *n‐*alkanes. The complementation of the mutant, conducted by introducing *smoABCD* in the genome as a single copy under a constitutive promoter or within a plasmid under a thiostreptone‐inducible promoter, allowed the recovery of the alkanotrophic phenotype as well as the capacity to degrade chlorinated *n*‐alkanes. The heterologous expression of *smoABCD* allowed a non‐alkanotrophic *Rhodococcus* strain to grow on pentane and *n*‐hexane when the gene cluster was introduced together with the downstream genes encoding alcohol and aldehyde dehydrogenases and a GroEL chaperon. BCP1 *smoA* gene was shown to belong to the group 6 SDIMOs, which is a rare group of monooxygenases mostly present in *Mycobacterium* genus and in a few *Rhodococcus* strains. SmoABCD originally evolved in *Mycobacterium* and was then acquired by *Rhodococcus* through horizontal gene transfer events. This work extends the knowledge of the biotechnologically relevant SDIMOs by providing functional and evolutionary insights into a group 6 SDIMO in *Rhodococcus* and demonstrating its key role in the metabolism of short‐chain alkanes and degradation of chlorinated *n*‐alkanes.

## Graphical abstract

In this study, we analysed a mutant of *Rhodoccocus aetherivorans* BCP1 characterized by a transposon insertion in the gene operon smoABCD encoding a soluble di‐iron monooxygenase of the rare SDIMO group 6. The smo mutant showed a reduced capacity to grow on propane, lost the ability to grow on butane, pentane and n‐hexane and was heavily impaired in the capacity to degrade chloroform and trichloroethane. Phylogenetic analyses showed that SmoABCD originally evolved in Mycobacterium and was then acquired by *Rhodococcus* through horizontal gene transfer events.

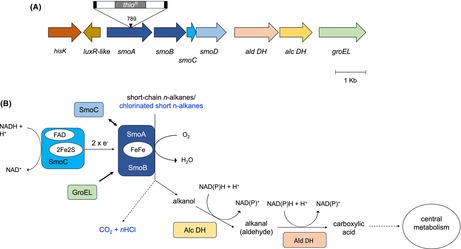

## INTRODUCTION

Alkanes are widely distributed in the environment as they are the most abundant hydrocarbons in crude oil (with an estimated abundance of 20%–50%). They are also naturally produced by living organisms (plants, algae and bacteria) as waste products, chemo‐attractants or for structural and defence purposes (Cappelletti, Fedi, & Zannoni, 2019). Short‐chain alkanes are important greenhouse gases, referring not only to methane but also to other gaseous alkanes (like ethane and propane) (Farhan Ul Haque et al., 2022). Due to their widespread presence in aquatic and soil ecosystems, bacterial oxidation of alkanes is a quite common process in natural environments and a major process in geochemical terms (van Beilen et al., 2003).

As alkanes are nonpolar and chemically inert, their utilization by microorganisms faces significant challenges concerning the energy required to activate them, their limited solubility, and their tendency to accumulate in cell membranes (Rojo, 2009). The study of alkanotrophic microbes (microbes that can utilize alkanes as growth substrate) and of the mechanisms allowing alkane degradation/metabolism is of great interest as the microbial degradation of *n*‐alkanes remains the main process for the remediation of oil‐contaminated areas (Brooijmans et al., 2009). Microbial growth on *n*‐alkanes can also induce co‐metabolic processes that allow the biodegradation of xenobiotics like halogenated hydrocarbons (Cappelletti et al., 2012, 2018; Holmes & Coleman, 2008).

In aerobic bacteria, the ability to utilize *n*‐alkanes as growth compounds is conferred by monooxygenases (MOs), a group of enzymes that catalyse the addition of one oxygen atom into organic compounds, activating the inert hydrocarbon molecule and forming an alcohol that is further oxidized and introduced in the central metabolism. Alkane hydroxylases involved in alkane metabolism typically belong to the alkane MO families AlkB, CYP153‐type cytochrome P450 and soluble di‐iron monooxygenases (SDIMOs). SDIMOs are multi‐component enzymes with wide applications in biodegradation and biocatalysis processes (Petkevičius et al., 2021). They are known to catalyse the initial oxidation of short‐chain alkanes, alkenes, halogenated alkanes, and aromatics (Coleman et al., 2006). Different SDIMOs are classified into different groups (1–6) based on gene organization, substrate specificity, and sequence similarity (Coleman et al., 2006, 2011; Holmes & Coleman, 2008; Leahy et al., 2003). While most of the knowledge on SDIMOs involved short‐chain *n‐*alkanes relies on studies of methane monooxygenases, SDIMOs involved in the metabolism of other short chain alkanes (gaseous and liquid *n‐*alkanes with carbon chain < C7) is less well understood. This catabolic activity is particularly abundant in the actinobacterial group CMNR including *Rhodococcus* together with the genera *Corynebacterium*, *Nocardia* and *Mycobacterium*.

Among alkanotrophic bacteria, members of the *Rhodococcus* genus are known to play an important role in the degradation of aliphatic hydrocarbons in natural environments and biotechnological settings like in enrichment bacterial mixed cultures exposed to alkanes as only carbon and energy source (Ciavarelli et al., 2012; Hamamura et al., 2006). *Rhodococcus* spp. strains are known to be able to transform a wide range of natural and xenobiotic compounds (Cappelletti et al., 2018; Ivshina et al., 2019; Larkin et al., 2005; Martínková et al., 2009; Orro et al., 2015; Presentato et al., 2018) in association with unique metabolic flexibility and high resistance/ tolerance to toxic compounds (Cappelletti et al., 2016; de Carvalho et al., 2014). The presence of large and complex genomes (up to 10.1 Mbp) together with catabolic gene redundancy is often considered the basis of the *Rhodococcus* catabolic versatility, functional robustness, adaptation to polluted and extreme environments and high‐performing environmental competition (Cappelletti, Zampolli, et al., 2019; Firrincieli et al., 2022). An increasing number of omics studies of microbial communities and alkanotrophic isolates from contaminated sites have extended the knowledge on the diversity of alkane MO in actinomycetes, mostly *Mycobacterium* and *Rhodococcus* (Coleman et al., 2006, 2011). On the other hand, the correlation between alkane MOs and xenobiotics co‐metabolism mostly involved only whole‐cell assays to determine biodegradation kinetic rates and enzymatic competition between the primary substrate (the alkane) and the co‐metabolized compound (e.g. halogenated hydrocarbons). Furthermore, only a few studies have applied molecular approaches to get insights into functional and regulatory aspects of the alkane MOs not only in *Rhodococcus* but also in other strains of CMNR group (Deng et al., 2018; Kotani et al., 2003; Martin et al., 2014; Sharp et al., 2007). This is mostly caused by the difficulties in genetic manipulation of these strains due to the presence of a robust mycolic acid‐containing cell wall, genomic instability, effective endogenous restriction systems that recognize unmethylated sites in exogenous DNA, the lack of universal molecular tools, high genome GC content (generally >60%) and low transformation and recombination efficiencies (Cappelletti, Zampolli, et al., 2019; Donini et al., 2021).

In this work, we analysed a mutant of *Rhodoccocus aetherivorans* BCP1 characterized by transposon insertion in the gene cluster *smoABCD* that encodes a monooxygenase belonging to group 6 SDIMOs. These are rare monooxygenases that have been scarcely studied, although they were previously suggested to be involved in co‐metabolic processes of recalcitrant pollutants (Cappelletti et al., 2015; Coleman et al., 2011; He et al., 2018). Here, we conducted growth and biodegradation assays and heterologous expression analyses to demonstrate for the first time the key role of a group 6 SDIMO in the metabolism of short‐chain *n*‐alkanes and degradation of chlorinated hydrocarbons. Phylogenetic and bioinformatic analyses also provide information on the evolutionary history of this SDIMO group that has been acquired by *Rhodococcus* from *Mycobacterium* through horizontal gene transfer (HGT) events.

## EXPERIMENTAL PROCEDURES

### Bacterial strains and culturing conditions

*Rhodococcus aetherivorans* BCP1 (DSM 44980) was initially isolated from a butane‐utilizing microbial consortium able to co‐metabolically degrade chloroform in batch slurry reactors (Frascari et al., 2006). *R. erythropolis* MTF was isolated from oil tailing ponds (Golby et al., 2014), while *R. erythropolis* SQ1 was a derivative strain of the type‐strain ATCC4277 with increased transformability (Dabbs et al., 1990). They were both found not to grow on short‐chain *n*‐alkanes in preliminary experiments. For the growth and degradation assays, *Rhodococcus* spp. strains and mutants were firstly pre‐cultured in 250 mL Erlenmeyer Baffled Flasks for 48 h at 30°C with shaking (150 rpm), containing 25 mL of LB medium [composed of (g L^−1^) NaCl, 10; Yeast Extract, 5; Tryptone, 10] supplied with the suitable antibiotic in the case of the mutants. The pre‐culture was then used to inoculate 50 mL of Mineral Salt Medium (MSM) supplied with the SL6 source of trace elements (as previously described by Presentato et al., 2018), at an initial OD_600_ of 0.05. Bottle microcosms (120 mL) containing 40 mL of MSM were incubated under shaking at 30°C and sealed with butyl rubber after the addition of alkanes at a final concentration of 0.1% (v/v) (Cappelletti et al., 2011). When necessary, antibiotics were added to select *Rhodococcus* transformants in the culture medium (thiostrepton at 10 μg/mL, tetracycline at 10 μg/mL and/or apramycin at 70 μg/mL). Bacterial growth was monitored by measuring optical density at 600 nm (OD_600_).

*Escherichia coli* DH5α was used as a host strain for the cloning of DNA fragments and the cells were cultivated in LB broth or on LB agar with ampicillin (50 μg/mL) when necessary. The bacterial strains and plasmids used in this work are listed in Table S1.

### DNA extraction and manipulation

Total DNA from *Rhodococcus aetherivorans* BCP1 and the transposon mutants was extracted from cells grown in 50 mL liquid LB cultures incubated at 30°C for 48 h (up to OD_600_ of 1–1.2). After cell harvesting, BCP1 WT and mutant cells were lysed using the protocol reported by Cappelletti et al. (2011), while standard molecular techniques were used for DNA manipulation and cloning (Sambrook et al., 1989). Shortly, for molecular cloning 1.5–3 μg of DNA were digested with appropriate restriction endonucleases (1 U) (Roche) for 3–4 h at 37°C. In multi‐enzyme digestion, compatible reaction buffers were used. Ligation reactions were performed overnight in a final volume of 15 μL using the T4 ligase (Roche) based on the manufacturer instructions except for the use of a temperature gradient ranging from 37°C to 4°C. Recombinant plasmids were introduced into *E. coli* DH5α using chemical transformation standard protocol (Sambrook et al., 1989). PCR and plasmid purifications were carried out using the Plasmid Mini Kit and PCR purification kit (Qiagen) according to the manufacturer's instructions. QIAquick Gel Extraction Kit (Qiagen) was used for the recovery and purification of DNA fragments excised from agarose gel.

### Generation of a transposon mutant library of *Rhodococcus aetherivorans* BCP1

Plasmid pTNR‐TA (Sallam et al., 2006) was used to construct the random transposon mutant library of *Rhodococcus aetherivorans* BCP1. pTNR‐TA is a transposable vector deriving from the non‐replicating transposon tool pTNR that carries the insertion sequence IS1415, a member of IS21 family initially identified in *R. erythropolis*. pTNR‐TA has a thiostrepton resistance gene (*thio* ^*R*^) as transposable‐marker gene and was introduced into *Rhodococcus aetherivorans* BCP1 cells through electroporation. Transformants were selected on the LB agar plates supplied with thiostrepton 5 μg/mL after 5 days of incubation at 30°C. The transformants were then re‐streaked on LB agar plates with thiostrepton 5 μg/mL for transposon insertion confirmation before being tested for their capacity to grow on C_6_ (0.1% v/v), C_16_ (0.1% v/v) or glucose (0.5% w/v) that were directly supplied on solid MSM plates as sole carbon and energy sources. Mutants that could not grow on glucose as the sole carbon source were identified as mutants that had transposon insertion in genes required for the growth on a minimal medium supplied with a single carbon and energy source and were omitted from subsequent analyses.

### Electroporation of *Rhodococcus aetherivorans* BCP1

To generate electrocompetent cells, *Rhodococcus* spp. strains cultures (BCP1, BCP1‐2.10 and MTF) were grown up to an OD_600_ of 0.6–0.7 in LB medium supplied with glycine 3.5% w/v, sucrose 1.8% w/v, isoniazid 150 μg/mL under shaking (150 rpm) at 30°C (around 18 h). The culture was then supplied with 3 μg of ampicillin and incubated for an additional 1.5 h at 30°C under shaking (150 rpm) before being centrifuged for 10 min at 6000 rpm at 4°C. The cell pellet was then washed once with 25 mL of ice‐cold EPB1 (20 mM Hepes pH 7.2, 5% glycerol) and twice with 10 mL of ice‐cold EPB2 (5 mM Hepes pH 7.2, 15% glycerol) before the final centrifuge at 6000 rpm for 10 min at 4°C. After discarding the supernatant, cells were suspended in 1–2 mL EPB2 and then aliquots of 200 μL were transferred into cold 1.5 mL tubes to be mixed with 1 μg of foreign DNA. After 5 min of incubation on ice, cell preparations were transferred in cold 0.2 cm‐cuvettes (Biorad) for electroporation. The electroporation was performed with Eporator (Eppendorf) using the setting 2.5 kV, 25 μF, and 400 Ω. Pulsed cells were immediately supplied with 1 mL of LB and transferred into a new tube. Cells were regenerated for 5–6 h with shaking at 150 rpm at 30°C, before being plated onto LB agar plates containing the appropriate antibiotic.

### Southern blotting

To confirm the single insertion of the transposon in the genomes of the mutant library, representative mutants were subjected to DNA extraction following the procedure previously described (Cappelletti et al., 2011). 5 μg of DNA was then subjected to enzymatic restriction with the enzyme PstI and ran on electrophoresis gel. The chromosomal DNAs from the agarose gels were then transferred to nylon membrane filters and Southern hybridization was performed according to the previously described protocol (Sambrook et al., 1989). The probe was made through PCR amplification using pTNR‐TA as template together with the primers TioR‐For (5′‐GGATCCGCCAGAGAGCGACGAC‐3′) and TioR‐Rev (5′‐CGCCTTCGAGGAGTGCCCG‐3′) to amplify part of the thiostrepton resistance gene that is comprised within the transposable region of the integrative vector. After PCR purification through Qiagen PCR cleaning kit, the DNA fragment was labelled with the DIG High Prime DNA labelling kit (Roche). Prehybridization and hybridization were carried out with DIG Easy Hyb (Roche) at 37°C overnight in a rotary oven. Washes were performed first in a 2× SSC (0.3 M NaCl plus 0.03 M sodium citrate)‐equivalent buffer for 5 min at room temperature, then in a 0.5x SSC‐equivalent buffer for 15 min at 65°C. The digoxigenin‐labelled probe was detected using CSPD (the alkaline phosphatase substrate) (Roche) as a chemiluminescent substrate according to instructions provided by the manufacturer.

### Inverse PCR (iPCR)

To identify the genes interrupted by the transposon insertion, total DNA was extracted and 1–2 μg samples were digested with *BglI*I or *Pst*I, purified with phenol/chloroform method and then EtOH precipitated. Digested DNA was then pooled in 15 μL milliQ H_2_O and then self‐ligated in a 30 μL reaction mixture at room temperature for 2 h. iPCR amplification was carried out using 2 μL of ligation mixture as the template, the TioseqTn1 (5′‐CAGTCATGGTCGTCCTACCG‐3′) and TioseqTn2 (5′‐CGAGGTATGTAGGCGGTGCT‐3′) as the primers and the BIOTAQ™ DNA polymerase (Bioline) using the manufacturer protocol. The PCR program was the following: initial denaturation at 95°C for 3 min; followed by 30 cycles of denaturation at 95°C for 50 s, annealing at 59°C for 50 s and extension at 72°C for 3 min, with a final extension at 72°C for 15 min. The PCR products were sequenced with the primer TioseqTn1.

### Plasmid construction for mutant complementation and heterologous expression

To carry out the complementation assay of the BCP1‐2.10 mutant strain, the mutant cells were transformed with two different constructs (visually displayed in Figure S1): (i) the self‐replicating *pTipQT1‐smoABCD* based on the shuttle vector pTipQT1 (Nakashima & Tamura, 2004a) and (ii) the transposable non‐replicating vector *pTNR‐AA/pNitsmoABCD* based on a modified version of the transposable tool pTNR‐TA (Sallam et al., 2007) (Table S1). To construct the plasmid *pTipQT1‐smoABCD*, the gene cluster *smoABCD* was PCR amplified from the BCP1 genome using the primers smoA‐NdeI‐For (5′‐TCACATATGACTACATCGGTCACAACTCAGCA‐3′) and smoD‐HindIII‐Rev (5′‐TGCAAGCTTTCACGCTGGGAGCTGGGCGGTTT‐3′) and the Taq polymerase Ex Taq (Takara) following manufacturer's instructions. After PCR clean‐up and restriction digestion with NdeI and HindIII, the *smoABCD* gene cluster was cloned in pTipQT1 within the compatible enzyme restriction sites NcoI‐HindIII. The pTipQT1 shuttle vector (Nakashima & Tamura, 2004a) contains a thiostrepton‐inducible promoter (P_tipA_), as well as the thiostrepton (*thio* ^*R*^) and tetracycline (*tet* ^*R*^) resistance genes for the selection in *Rhodococcus* and the ampicillin (*amp* ^*R*^) resistance cassette for the selection in *E. coli*. To construct the plasmid *pTNR‐AA/pNitsmoABCD*, the thio^r^ cassette in pTN‐TA was replaced with the apramycin (*apra* ^*R*^) resistance gene. The *apra* ^*R*^ gene was first PCR amplified from pIJ8600 (Takano et al., 1995) using the primers Apra‐BamHI‐For (5′‐CTCAGGATCCTCTGACGCTCAGTGGAAC‐3′) and Apra‐HindIII‐Rev (5′‐TCACAAGCTTACGTCGCGGTGAGTTCAG‐3′) and then cloned in pTNR‐TA within the restriction enzyme sites BamHI and HindIII (replacing *thio* ^*R*^). The gene cluster *smoABCD* amplified with the primers smoA‐NdeI‐For and smoD‐HindIII‐Rev was inserted within the restriction enzyme sites NcoI‐ HindIII in the plasmid pNitQT1 (Nakashima & Tamura, 2004b) before being amplified with the primers pNit‐StuI‐For (5′‐ACTCATATGTCACGCTGGGAGCTGGGCGGTT‐3′) and smoD‐HindIII‐Rev to obtain the pNit‐*smoABCD* fragment corresponding to the *smoABCD* operon under the control of the constitutive P_nit_ promoter. The PCR product was then inserted into pTNR‐AA within the StuI‐HindIII restriction sites, to finally obtain pTNR‐AA/*pNitsmoABCD*, whose genomic insertional event inserts pNitsmoABCD together with *apra* ^*R*^.

For complementation experiments, *R. erythropolis* MTF was transformed using *pTipQT1‐smoABCD* and an additional construct including the entire gene cluster *smoABCD/aldDH/alcDH/groEL* (*smoABCD* together with the alcohol DH, aldehyde DH and GroEL chaperonin) (Table S1). To generate the construct for heterologous expression, the gene cluster *smoABCD/aldDH/alcDH/groEL* was amplified from the BCP1 genome by using the primers smoA‐NdeI‐For (5′‐TCACATATGACTACATCGGTCACAACTCAGCA‐3′) and groEL‐SpeI‐Rev (5′‐ATCACTAGTATCAGAGGGGAGGCATTTTCGA‐3′) using the Taq polymerase Ex Taq (Takara) following manufacturer's instructions. After PCR purification and enzymatic restriction digestion, the *smoABCD/aldDH/alcDH/groEL* gene cluster was cloned in the vector pTipQT1 within the enzymatic restriction sites NdeI‐SpeI to construct the vector pTipQT1‐*smoABCD/aldDH/alcDH/groEL*.

The constructs containing *smoABCD* and *smoABCD/aldDH/alcDH/groEL* were first obtained in *E. coli* DH5α and then the constructs and the empty vectors were transformed in *Rhodococcus* spp. cells by electroporation. *Rhodococcus* cells transformed with pTipQT plasmids were selected on LB agar plates supplied with tetracycline at a final concentration of 10 μg/mL.

### Alkane and chlorinated alkane degradation assays

For the biodegradation assays, resting cell experiments were performed by growing first the bacterial cells on an inducer alkane and then testing the biodegradation activity in phosphate buffer. In particular, after the pre‐inoculum in LB and the cell washing with phosphate buffer (NaH_2_PO_4_ 7.72 g/L, Na_2_HPO_4_ 20.44 g/L, pH 7.2), an aliquot of cell suspension was used to inoculate 20 mL liquid MSM in 119 mL bottles that were then closed with rubber caps and sealed with metal rings, before being supplied with propane (the only alkane that allows the growth of the BCP1‐2.10 strain) as sole carbon and energy source. These cultures were then incubated for 168 h at 30°C, while shaking at 150 rpm. At the end of bacterial growth, cells were pelleted and washed once with 10 mL phosphate buffer. The biomass pellets were then resuspended in phosphate buffer to obtain an OD_600_ of 0.2–0.3. Before biomass addition, 12.3‐mL vials were pre‐filled with 1 mL of sterile phosphate buffer together with each substrate under analysis, then closed with Teflon caps (to limit the abiotic loss of chlorinated alkanes), sealed with metal rings and finally incubated for 1 h at 30°C for gas–liquid phase equilibrium. In each bottle, the following substrates were added separately using sterile syringes at the following concentration: propane 70 μM, butane 55 μM, pentane 12 μM, hexane 7 μM, chloroform (CF) 2 μM and 10 μM, 1,1,2‐trichloroethane (1,1,2‐TCA) 10 μM and 50 μM. After the first gas chromatography analysis (T_0_), the biomass aliquots (500 μL) were inoculated in the bottles, which were then incubated at 30°C and 150 rpm and monitored at different time points from the initial GC measurement (2.5, 5, 20 and 51 h). Biodegradation of *n*‐alkanes and chlorinated alkanes was assessed through gas chromatography by following the procedures described by Frascari et al. (2006). Specific biodegradation rates were calculated by considering the mass of compound degraded normalized over the amount of cellular biomass measured as mg of proteins. For protein quantification, 200 μL of the same cell suspension used to inoculate the 12.3 mL vials was analysed through Lowry assay using bovine serum albumin (BSA) as standard (Lowry et al., 1951).

### Phylogenetic analysis to identify the SDIMO family of SmoABCD

Phylogenetic analysis of *Mycobacteriaceae* sDIMO was performed as follows. Non‐redundant protein accession of the Methane/Phenol/Toluene Hydroxylase (MPTh) protein family belonging to Actinobacteriota was retrieved from the NCBI Protein database at the following link: https://www.ncbi.nlm.nih.gov/protein/?term=%28%232%29+AND+%22actinobacteria%22%5Bporgn%3A__txid201174%5D+. The protein accessions (WP_*) were then used to retrieve all the unique SDIMO protein sequences that were associated to Actinobacteriota phylum from NCBI Identical Protein Group (IPG) database. The Actinobaceriota Assembly accessions obtained from the IPG database were finally filtered to keep only genomes and Methane/Phenol/Toluene Hydroxylase protein affiliated to the *Mycobacteriaceae* family according to the Genome Taxonomy Database v214. The identification and classification of the MPTh alpha subunits into group 1, group 2, group 3, group 3‐like, group 4, group 5 and group 6 were then carried out via phylogenetic analysis using as reference database the alpha‐subunit data set described in Zou et al. (2021). Briefly, the Zhou alpha‐subunit data set and *Mycobacteriaceae* MPTh were aligned between each other with MAFFT (Katoh et al., 2002) in auto mode (−‐auto) and the resulting alignment file was trimmed with TrimAl (Capella‐Gutiérrez et al., 2009) in automated mode (−automated1). The trimmed file was used to construct an unrooted maximum likelihood phylogenetic tree in FastTree (Price et al., 2009) with default parameters. The resulting phylogenetic tree was visually inspected to identify and classify the MPTh alpha subunits. Finally, a definitive maximum likelihood phylogenetic tree representing the *Mycobacteriaceae* MPTh alpha subunits was constructed in IQ‐TREE v.2.2.2.6 (Nguyen et al., 2015) using the Q.yeast+F + I + G4 substitution model.

### Gene tree reconciliation analysis

GeneRax (Morel et al., 2020) was used to identify horizontal gene transfer events involving the alpha subunits of the group 6 SDIMOs between members of *Mycobacteriaceae* family. For this purpose, a phylogenetic tree representing the group 6 and group 3 alpha subunits (group 3 was included to generate a rooted binary tree) was reconciled against the GTDBtk‐based species tree of the *Mycobcateriaceae* family using the UndatedDTL probabilistic model for computing the reconciliation and the SPR tree search mode. The resulting transfer events from the *Mycobacterium* donor strain and within members of the *Rhodococcus* genus were finally visualized in iToL (https://itol.embl.de/).

## RESULTS

### Isolation and genetic characterization of a transposon insertion mutant of *R. aetherivorans* BCP1 defective in the growth on short‐chain *n*‐alkanes

**FIGURE 1:**
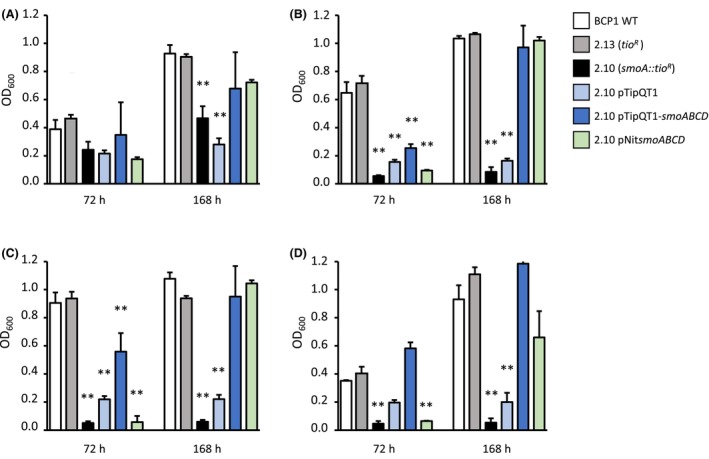
. Growth assays of *R. aetherivorans* BCP1 on short‐chain *n*‐alkanes. Growth was measured as OD_600_ values at two different time points (72 and 168 h) after the inoculation on minimal medium (MSM) supplied with C_3_ (A), C_4_ (B), C_5_ (C) or C_6_ (D) as only carbon and energy source. Results shown are means ± standard deviation with *n* = 3. Asterisks indicate statistically different groups respect BCP1 wild type (BCP1 WT) according to one‐way ANOVA analysis. *p < 0.5, **p < 0.01.

A library of random insertional mutants of *R. aetherivorans* BCP1 was created and screened for the capacity to grow using *n*‐alkanes as the only carbon and energy source. A non‐auxotrophic mutant, named BCP1 2.10, was selected for the inability to grow on a solid minimal medium supplied with *n*‐hexane (C_6_). Conversely, this mutant retained the capacity to grow on *n*‐hexadecane (C_16_) and on other standard carbon sources (Figures S2A–C and S3). This initial screening suggested this mutant was deficient in the ability to grow on liquid short‐chain *n*‐alkanes. To characterize further BCP1 2.10, we extended the range of tested *n*‐alkanes by inoculating the mutant strain in liquid cultures with propane (C_3_), butane (C_4_), pentane (C_5_) or hexane (C_6_) as only carbon and energy source (Figure 1A–D). In these growth assays, in addition to the WT strain, the insertional mutant BCP1 2.13 was also utilized as a positive control as it was not compromised in the ability to grow on *n*‐alkanes and carried *thio* ^*R*^ gene, thus evaluating the possible influence of antibiotic resistance gene on the bacterial growth. As a result of the growth assays, BCP1 2.10 almost completely lost the capacity to grow on C_4_, C_5_ and C_6_ (Figure 1). On the other hand, 2.10 showed a certain capacity to grow on C_3_ although the growth performance was significantly lower than the controls (WT and 2.13 mutant strains). Mutant 2.10 maintained the capacity to grow on the alkanes' metabolic intermediates 1‐propanol, 1‐butanol and 1‐hexanol (Table S2), indicating that the mutation affected the first alkane oxidation reaction and not the subsequent oxidation steps leading to the conversion of the alcohol into aldehyde.

**FIGURE 2:**
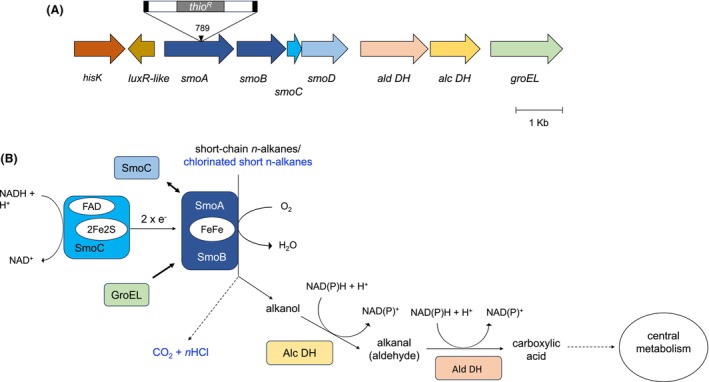
The soluble di‐iron monooxygenase (SDIMO) Smo from *Rhodococcus aetherivorans* BCP1. (A) Schematic organization of the gene cluster *smoABCD* encoding the soluble di‐iron monooxygenase (SDIMO) Smo and the flanking regions that are co‐localized on the megaplasmid pBMC2 in *R. aetherivorans* BCP1 genome. Gene are displayed as arrows. The single genes of the smoABCD operon code for smoA, the monooxygenase alpha subunit, smoB, the monooxygenase beta subunit, *smoC* the coupling protein, smoD, the reductase. The flanking genes code for *ald DH*, the aldehyde dehydrogenase (DH), *alc DH* the alcohol DH, *groEL* GroEL the chaperonin, *hisK* the histidine kinase, *luxR‐like* the cognate response regulator. The transposable element of pTNR‐TA that interrupt *smoA* gene in the mutant 2.10 is shown together with the 1070 nt‐long thiostrepton resistance gene (*thio* ^*R*^) and the insertion site (at the nucleotide 789 of *smoA*). The locus tags of the genes go from N505_0128845 (for *hisK*) to N505_0128885 (for *groEL*) B. (B) Cofactor composition of the Smo components and predicted enzymatic reactions catalysed by Smo, Alc DH and Ald DH. The metabolic pathway and reaction intermediates for the alkanes are typed in black, the reaction involved in the degradation of chlorinated alkanes are in blue. Dashed lines correspond to more reactions.

The single insertion of pTNR‐TA into the BCP1 genome was confirmed through the Southern blot analysis of several library mutants, including 2.10 and 2.13 (Figure S4). The genetic characterization of the mutant BCP1 2.10 was then performed by inverse PCR. As a result, the strain was found to be mutated at the level of the *smoA* gene (*smoA*::*tio* ^*R*^) that is included within the *smoABCD* operon encoding a soluble di‐iron monooxygenase (SDIMO) (Cappelletti et al., 2015). In particular, the transposon was found to interrupt the *smoA* gene at the level of the nucleotide 798 from the ATG start codon and two 9 bp direct repeats ‘GACAATTTC’ flanked the transposon insertion, as displayed in Figure 2A. As the transposon insertion did not shift the reading frame, no polar effects are expected on the expression of adjacent genes.

### Complementation of the smoA mutant BCP1‐2.10 strain

The complementation of the smoA mutant 2.10 strain was carried out by transforming the mutant with two plasmid systems, separately, (i) the replicative plasmid pTipQT1 harbouring the *smoABCD* gene cluster under the control of the P_tipA_ thiostrepton‐inducible promoter (BCP1 2.10 pTipQT1‐*smoABCD*), (ii) the non‐replicating plasmid pTNR‐AA/*pNitsmoABCD* harbouring the *smoABCD* operon under the constitutive promoter P_nit_ (Table S1). While the complementation with the first plasmid is expected to lead to the introduction of 40–60 copies of *smoABCD* inside the cell that are contemporary expressed at high level because under the inducible promoter (pTip) (Nakashima & Tamura, 2004a), the use of the non‐replicative plasmid is associated with the expression of only one single gene copy that is integrated in the genome under the control of a constitutive promoter (pNit) that has similar expression levels to pTip (Nakashima & Tamura, 2004b). Both the complemented strains were tested for the phenotype rescue by analysing the growth in liquid cultures in the presence of C_3_, C_4_, C_5_ and C_6_ as only carbon and energy sources. As a result, both 2.10 pTipQT1‐*smoABCD* and 2.10 *pNitsmoABCD* strains showed a complete recovery of the growth capacities after 168 h of incubation (Figure 1). In the case of the strain 2.10 pTipQT1‐*smoABCD*, the phenotype reacquisition was observed even after 72 h of growth, suggesting that the number of *smoABCD* copies and/or the control by the inducible promoter pTip, positively impacted the growth on *n‐*alkanes. The control strain harbouring the only plasmid (2.10 pTipQT1) could grow on alkanes slightly better than the mutant 2.10. A possible hypothesis about this behaviour regards the influence that the antibiotic resistance gene *tet* ^*R*^ (on pTipQT1) might have on global bacterial metabolism (Martínez & Rojo, 2011). All the complemented mutant strains showed the same growth when a standard carbon and energy source like glucose was added (Figure S3), demonstrating the specific role of the Smo protein on alkane metabolism.

### Heterologous expression of *smoABCD* provides a *Rhodococcus* strain with the ability to grow on short‐chain *n*‐alkanes

**FIGURE 3:**
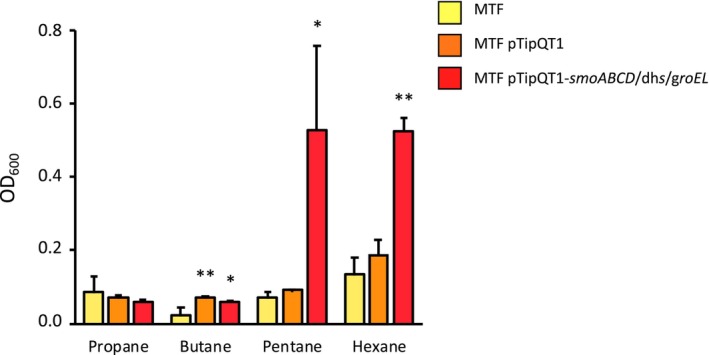
Heterologous expression of smoABCD in *R. erythropolis* MTF. Growth of MTF wild type (MTF) and MTF transformed with pTipQT1‐*smoABCD/dhs/groEL* (MTF pTipQT1‐*smoABCD/dhs/groEL*) or the empty vector (MTF pTipQT1) is shown as OD_600_ values measured after 72 h of culture in minimal medium (MSM) supplied with C_3_, C_4_, C_5_ and C_6_ (0.1% v/v) as only carbon and energy source. Results shown are means ± SD. Statistically significant differences (using one‐way ANOVA analysis) respect to the MTF wild type are shown with asterisks *p < 0.05, **p < 0.01.

To further investigate the role of SmoABCD in bacterial growth on short‐chain *n*‐alkanes, the heterologous expression of the monooxygenase gene cluster was performed using the two different *Rhodococcus* strains *R. erytropolis* MTF and *R. erytropolis* SQ1. These bacterial strains are unable to grow on short‐chain *n*‐alkanes (C_3_–C_6_) and were transformed via electroporation with the empty pTipQT1 vector, the pTipQT1 containing the only *smo* gene cluster (*smoABCD*), and the pTipQT1 containing the *smoABCD* together with the genes that in BCP1 genome are immediately downstream the cluster, which are the alcohol and aldehyde dehydrogenase genes and the GroEL chaperone gene. The results of the growth assays on liquid minimal salts medium supplemented with Thio and different short‐chain *n*‐alkanes, showed that the introduction of pTipQT1‐*smoABCD* did not allow either of the two strains to grow on short‐chain alkanes (data not shown). Conversely, *R. erytropolis* MTF (but not *R. erytropolis* SQ1) transformed with pTipQT1‐*smoABCD/aldDH/alcDH/groEL* acquired the ability to grow on C_5_ and C_6_ (Figure 3; Figure S5). This result demonstrated on one hand that the expression of the SDIMO Smo provides host strain with the ability to grow on some short‐chain *n*‐alkanes, and on the other that the genes involved in protein folding and in enzymatic reactions downstream of the initial alkane oxidation step are necessary for the alkane metabolism mediated by the SDIMO Smo.

### Role of the Smo in the degradation of short‐chain alkanes and chlorinated aliphatic alkanes (ChlA) in *R. aetherivorans* BCP1

**FIGURE 4:**
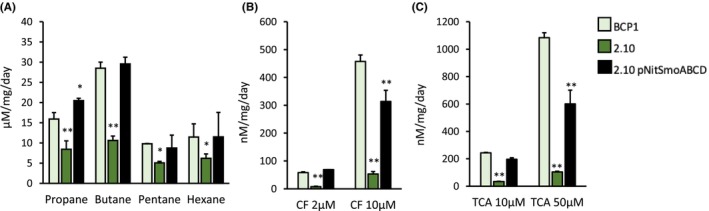
Role of the monooxygenase Smo in the degradation of short‐chain n‐alkanes and chlorinated alkanes in *Rhodococcus aetherivorans* BCP1. The capability of the wild‐type strain BCP1 (BCP1), the mutant 2.10 strain (2.10) and the complemented mutant strain (2.10 pNitsmoABCD) to degrade short chain alkanes (each supplied in MSM at 0.1% v/v) (A) and chlorinated *n*‐alkanes (chloroform, CF, and 1,1,2‐trichloroethane, TCA, each supplied at the concentrations 2 and 10 μM) (B, C) are shown in terms of specific degradation rates (i.e. concentration of the compound degraded per mg of cellular proteins per day). Asterisks indicate statistical significance (one‐way ANOVA analysis compared to BCP1). *p < 0.05, **p < 0.01.

In order to define the role of monooxygenase Smo in the degradation of alkanes and in the co‐metabolism of chlorinated alkanes in BCP1, the mutant strain 2.10, the complemented strain 2.10 pNit*SmoABCD* and the WT strain were grown on propane and then exposed to short chain alkanes and chlorinated alkanes under resting cell conditions. Propane was used since it is the only short‐chain alkane that allowed the growth of the 2.10 mutant (although at a lower rate, Figure 1). This was needed to develop cell biomass of the 2.10 mutant with the same alkane‐induced enzymatic asset (needed for chlorinated hydrocarbon co‐metabolism) of the BCP1 WT strain except for a functional SDIMO SmoABCD. The cell growth of the 2.10 mutant could be attributed to the presence of the SDIMO PrmABCD that is transcriptionally induced at high levels by propane (Cappelletti et al., 2015). The activity of SmoABCD could be therefore provided by the difference observed between the WT strain (expressing both the SDIMOs SmoABCD and PrmABCD) and the 2.10‐BCP1 strain (expressing the only SDIMO PrmABCD) in terms of biodegradation performance. The biodegradation assay results are reported in Figure 4A,B which displays the specific degradation rates for all the tested compounds considering the concentration of the substrate degraded over time normalized over the biomass amount, to avoid interferences due to lower 2.10‐BCP1 biomass growth. As a result, the mutant strain 2.10 showed impaired capacity to degrade C_3_, C_4_, C_5_ and C_6_ as compared to the WT strain. Nevertheless, 2.10 did not completely lose the capacity to degrade any of these substrates, probably due to the presence of an intact Prm monooxygenase in the 2.10 mutant that can metabolize, to some extent, the tested substrates. The analysis of the complemented mutant strain, 2.10 pNit*SmoABCD*, showed that the expression of the Smo monooxygenase allowed recovery of the phenotype of growth on all the short alkanes even showing improved performance in the degradation of C_3_ (Figure 1). These results demonstrate the role of Smo monooxygenase in the degradation performance of BCP1 towards all the short alkanes under analysis.

The analysis of the degradation of chlorinated alkanes showed that the absence of the Smo monooxygenase in mutant 2.10 strongly impaired the degradation rate of both chloroform (CF) and 1,1,2‐trichloroethane (TCA) (Figure 4B). The complementation of the mutant with a functional Smo monooxygenase allowed for a complete recovery of the degradation performance when the chlorinated alkanes were added at the lowest concentration under analysis. The complemented mutant showed an improvement but not a complete regain of the function when the chlorinated alkanes were tested at higher (10 μM) concentration. These results demonstrate a major role of Smo monooxygenase in the co‐metabolism of CF and TCA and a possible influence of the expression apparatus/regulatory mechanisms when high concentrations of the chlorinated alkanes are tested. Indeed, the *smoABCD* cluster is under the control of an inducible promoter in the WT strain, while the complemented strain has the Smo function under the control of the constitutive promoter pNit. The possible difference in Smo monooxygenase production in the two strains might explain the incapacity of the complemented strain to degrade the chlorinated alkanes with the same degradation rate as the WT one.

### Phylogenetic analysis of the alpha subunit of the SDIMOs from *R. aetherivorans* BCP1

**FIGURE 5:**
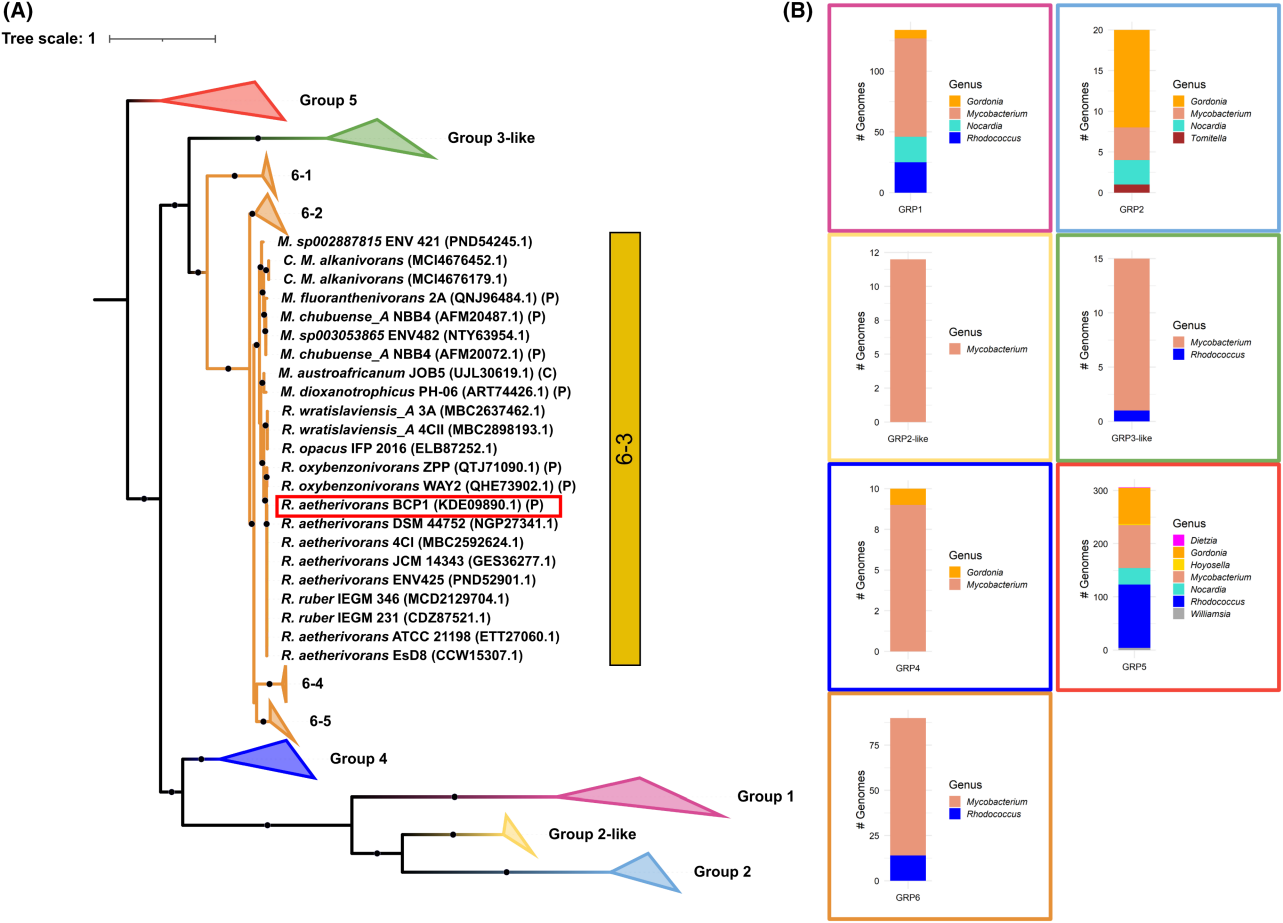
Maximum likelihood phylogenetic tree and distribution of the SDIMO groups (from 1 to 6) of *Mycobacteriaceae* family with an enlargement on the SDIMO group 6 sub‐clusters (from 6‐1 to 6‐5). (A) Phylogenetic distances in the phylogenetic tree were calculated using the Q.yeast+F + I + G4 substitution model. Ultrafast bootstrap support values >80 (1000 bootstrap replicates) are indicated as black dots. *R. aetherivorans* BCP1 SmoA is framed by a red box. Besides each strain name, NCBI protein accession is indicated between brackets together with genomic location, if known, corresponding to P, for plasmid or C, for chromosome. (B) The distribution of each SDIMO group among different *Mycobacteriaceae* genera is shown by framing each bar plot in boxes that are coloured in the same way as the phylogenetic tree.

Initial orthologous analyses of the BCP1 SmoA protein carried out through BLAST search in the NCBI non‐redundant protein database, showed that this gene has significant similarity (>70%) only with alpha subunits of SDIMOs from strains of *Rhodococcus* and *Mycobacterium* genera of *Mycobacteraceae* family (Table S3). We therefore conducted a phylogenetic analysis including all the SDIMO alpha subunits carried by bacterial strains of the *Mycobacteriaceae* family strains present in the database to define the SDIMO family classification of BCP1 SmoA and to get insights into the evolutionary history of this biotechnologically important group of enzymes (Figures 5 and S6; Table S4). The phylogenetic tree showed the clustering of SDIMOs in 7 main groups, that is, group 1, group 2, group 2‐like, group 3‐like, group 4, group 5 and group 6. The clustering of the SDIMOs shows that the group 6 falls in a monophyletic clade together with the alpha subunits of SDIMOs group 3‐like and share a common ancestor with SDIMOs of group 1, group 2‐like and group 4. Conversely, SDIMOs of group 5 (including the BCP1 PrmA protein) belong to a separate clade that is composed of alpha subunits retrieved from different genera demonstrating a more widespread distribution among actinobacterial lineages despite the high sequence similarity (Figure S6).

The phylogenetic analysis clustered the BCP1 PrmA protein within the SDIMOs group 5, and the BCP1 SmoA protein within the SDIMO group 6 together with other *smoA* from several *Mycobacterium* spp. strains and a few *Rhodococcus* spp. strains (Figures 5 and S6). The SDIMOs group 6 was shown to be in turn sub‐grouped into 5 clusters (bootstrap support >80%) with the BCP1 SmoA included in the only sub‐cluster (i.e. sub‐group 6–3) comprising other alpha subunits from several *Rhodococcus* species (*R. aetherivorans*, *R. ruber*, *R. oxybenzonivorans*, *R. wratislaviensis* and *R. opacus*) together with the *Mycobacterium* species *M. alkanivorans*, *M. dioxanotrophicus*, *M. sp002887815*, *M. chubuense*, *M. fluoroanthenivorans*, *M. sphagni*, *M. austroafricanum*. All the other 4 sub‐clusters of the SDIMO group 6 included only *Mycobacterium* species (sub‐groups 6–1, 6–2, 6–4 and 6–5 in Figures 5 and S6; Table S4). Interestingly, the group 6 SDIMO alpha subunits SmoA detected in *Mycobacterium* complete genomes showed both chromosomal and plasmidic location, while in *Rhodococcus*, complete genomes *smoA* genes were exclusively located in plasmids (Figure 5; Table S4). On the other hand, all *prmA* genes (group 5 SDIMOs), including the one from BCP1, had chromosomal localization. Gene cluster analysis of the group 6 *Rhodococcus*‐*Mycobacterium* sub‐cluster 3 revealed complete conservation of the *smoABCD* coding genes, while the regulatory genes *hisK* and *luxR‐like*, and the accessory genes *groEL*, *alc DH* (encoding an NDMA‐dependent alcohol dehydrogenase) and *ald DH* (encoding an aldehyde dehydrogenase) were only partially conserved because missing in some of *Mycobacterium* strains carrying the sub‐cluster 6–3 *smoA* genes (Figure S7).

**FIGURE 6:**
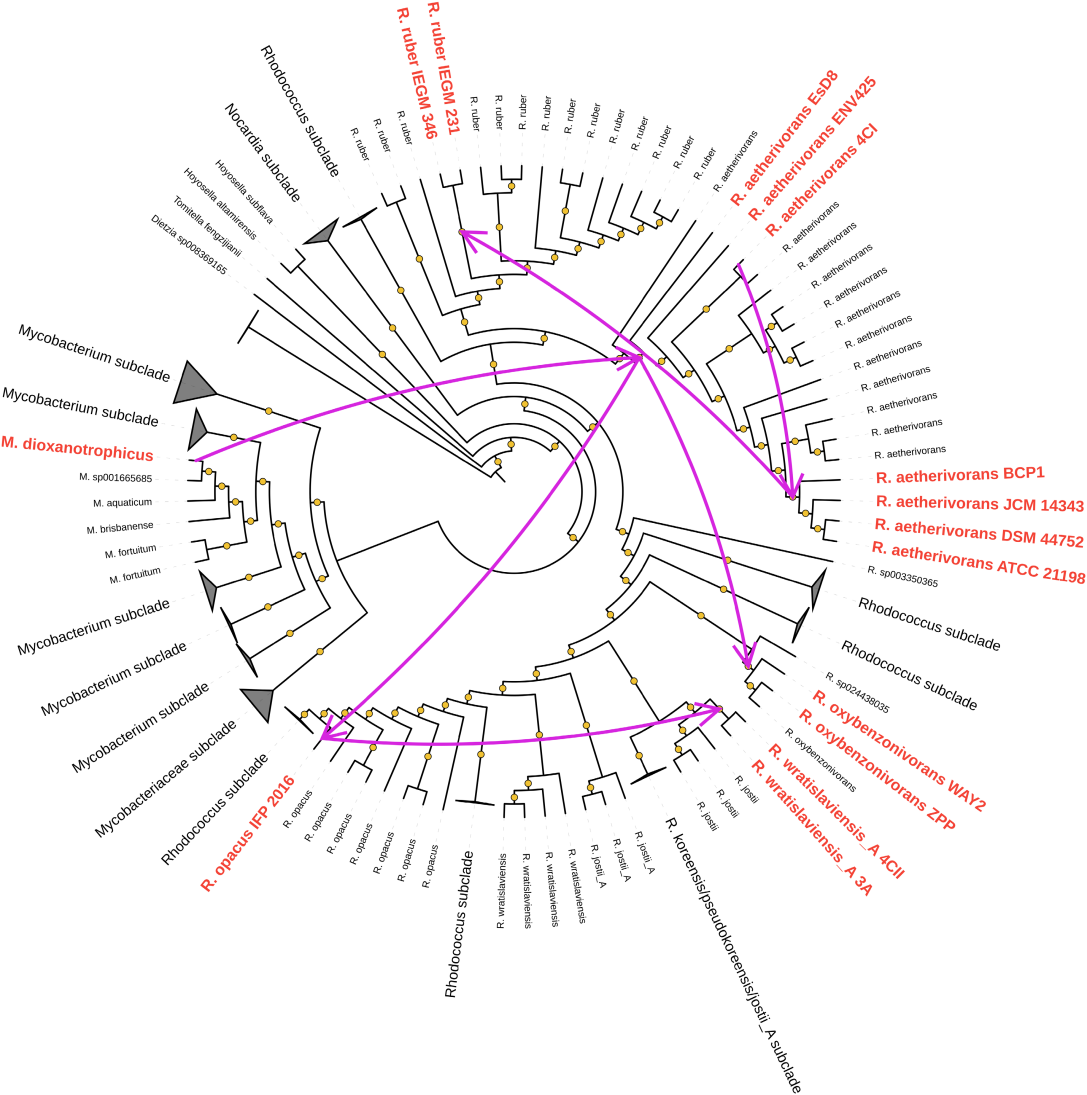
Predicted GeneRax horizontal gene transfer (HGT) events of group 6 SDIMOs between members of *Rhodococcus* and *Mycobacterium* (only these bacterial genera possess group 6 SDIMOs). HGT events occurring between *Rhodococcus* spp. strains are shown as pink arrows. Phylogenetic distances were calculated using the WAG substitution model. Ultrafast bootstrap support values >80 (1000 bootstrap replicates) are indicated as yellow dots.

A gene tree/species tree reconciliation analysis was then performed to elucidate the phylogenetic history of the *smoA* genes in BCP1 and, more in general, in the *Rhodococcus* genus. From the analysis of the transfer events is clear that *M. dioxanotrophicus* is the donor bacterial species of the group 6 SDIMOs in the *Rhodococcus* genus with the recipient represented by the *R. aetherivorans* lineage. Later, *R. aetherivorans* was the donor species of the group 6 SDIMOs in strains of the *R. opacus* (IFP 2016), *R. ruber* (IEGM 231 and 346), *R. oxybenzonivorans* (WAY2 and ZPP), *R. wratilasviensis_A* (4CII and 3A) lineages (Figure 6). This reconciliation analysis together with the plasmidic localization of *smoA* in *Rhodococcus* strains suggests that group 6 SDIMO in *Rhodococcus* appeared for the first time in the *R. aetherivorans* lineage through a horizontal gene transfer involving *M. dioxanotrophicus* as the donor bacterial species.

## DISCUSSION

In this work, we first demonstrate the role of a group 6 soluble di‐iron monooxygenase (SDIMO) in the metabolism and degradation of gaseous and short‐chain liquid *n‐*alkanes and chlorinated alkanes (chloroform and 1,1,2‐trichloroethane) by analysing a transposon mutant strain of *Rhodococcus aetherivorans* BCP1. Previously, apart from the methane monooxygenases (group 3 SDIMOs) and soluble butane monooxygenase of *Thauera butanovora*, the only studies that characterized mutants of SDIMOs associated with *n‐*alkane metabolism targeted (i) the SDIMO group 5 (*prmABCD* gene cluster) that was found to be fundamental for the growth on propane by *Mycobacterium*, *Rhodococcus*, and *Gordonia* strains (Kotani et al., 2003; Sharp et al., 2007), (ii) the SDIMO group 3‐like in a *Rhodococcus* strain that was reported to play a key role in the growth on ethane and propane (Zou et al., 2021). The *R. aetherivorans* BCP1 mutant (2.10 mutant) that we analysed in this study has a transposon insertion in the gene *smoA* coding for the large subunit of the hydroxylase component of the SDIMO SmoABCD (group 6 SDIMO). The mutant was found to have a reduced growth on propane, while it almost completely lost the capacity to grow on butane, pentane and *n‐*hexane. These results are in line with a previous study that reported the over‐expression of the *smoABCD* gene cluster in BCP1 cells exposed to diverse gaseous and liquid short‐chain *n‐*alkanes (Cappelletti et al., 2015).

In addition to the phenotypic analyses of the mutant strain, the heterologous expression of *smoABCD* conducted in a non‐alkanotrophic *Rhodococcus* host strain provided the capacity to grow on pentane and hexane when the gene cluster was introduced together with the flanking genes encoding an alcohol dehydrogenase (DH), an aldehyde DH and a GroEL chaperonin. This result demonstrates the possibility to transfer the metabolic function associated with this SDIMO in other *Rhodococcus* strains with biotechnological relevance to create recombinant strains for in situ or ex situ bioremediation applications. Furthermore, it demonstrates the key role of the genes flanking *smoABCD*. This is in line with the positional conservation that included not only *smoABCD* but also the downstream genes encoding an aldehyde DH, an alcohol DH and a GroEL chaperon (Figure S7). The two DHs are thought to catalyse the oxidation reactions that convert the alkanol (produced by Smo) into carboxylic acid which enters the central metabolism (Figure 2B). Conversely, the GroEL chaperonin might have a fundamental function in the correct folding of the hydroxylase subunit of SmoABCD (Furuya et al., 2003; McCarl et al., 2018). In line with these results, the same aldehyde DH and the alcohol DH were previously found to be over‐expressed in the proteome of BCP1 cells grown on butane and hexane (Cappelletti et al., 2015). Furthermore, the co‐expression of a chaperonin‐like gene was reported to be necessary for the activity of other SDIMOs expressed in host strains (Furuya et al., 2003; Kotani et al., 2003; Kurth et al., 2008; Stafford et al., 2003).

The recombinant *Rhodococus* strain carrying *smoABCD/aldDH/alcDH/groEL* gene cluster could grow using pentane and hexane, but it could not grow on the gaseous *n‐*alkanes butane and propane. Possible hypotheses concern the absence of additional genetic functions in the host strain that are needed for effective use of gaseous alkanes or their oxidation products as carbon sources (including enzymes involved in the metabolism of the alcohols and aldehydes produced by the first alkane oxidation). Furthermore, heterologous expression of Smo provided *n‐*alkane growth capacity to one of the two non‐alkanotrophic *Rhodococcus* strains that were tested in this study (to *R. erythropolis* MTF but not to *R. erythropolis* SQ1). Although genome‐based indications are not available for the two strains, it is known that different *R. eryhtropolis* strains can have wide catabolic and genetic diversity (de Carvalho & da Fonseca, 2005). Therefore, this result might be ascribed to the presence in MTF strain (but not in SQ1) of specific genetic functions possibly associated to alkane uptake, oxidation products metabolism and/or mechanisms supporting resistance to the toxicity of liquid short‐chain *n‐*alkanes (Cappelletti, Fedi, & Zannoni, 2019; de Carvalho & da Fonseca, 2004).

The mutation of the group 6 SDIMO Smo heavily impaired the biodegradation capacities of *R. aetherivorans* BCP1 not only towards short‐chain *n‐*alkanes but also towards the chlorinated hydrocarbons chloroform and trichloroethane. The biodegradation experiments were conducted using resting cells that were grown on propane before being tested in whole‐cell assays. This is probably the reason why the mutation of *smoA* did not completely erase biodegradation capacities towards the tested *n‐*alkanes. Indeed, BCP1 cells grown on propane are known to express the other SDIMO gene cluster *prmABCD* (Cappelletti et al., 2015). After being produced, the SDIMO Prm could be able to recognize and oxidize butane, pentane and *n*‐hexane although they are not the primary substrates/inducers thanks to a certain level of aspecificity that is known for SDIMO enzymes, allowing the co‐oxidation of structural analogues (Coleman et al., 2006; McDonald et al., 1997; Sluis et al., 2002). On the other hand, group 6 SDIMO seems to have a major role in the degradation of chloroform and trichloroethane as its mutation determined a 90% decrease in biodegradation rate.

Therefore, alkane and chlorinated hydrocarbon metabolism in BCP1 involve the activity of the two SDIMOs Smo and Prm which have partly overlapped alkane substrate specificity and inducer range but different degradation kinetics. A third alkane monooxygenase that belongs to the AlkB family also participates in alkane metabolism in BCP1 by targeting *n*‐alkanes from C_6_ to C_32_ (Cappelletti et al., 2011). This alkane range also partially overlaps with the one associated with *smoABCD* (C_6_, C_7_ and C_8_ induce both Smo and AlkB), suggesting a specificity of the different monooxygenases that is related to the alkane chain length. The coexistence of multiple alkane hydroxylase genes in *Rhodoccocus* could be the result of evolution processes aimed at differentiating and widening the catabolic processes of members of this genus in response to the presence of specific classes of hydrocarbons.

The phylogenetic and comparative analyses of the alpha subunit of the *smoABCD* and *prmABCD* gene clusters classified the two BCP1 SDIMOs into groups 6 and 5, respectively, which show distinct evolutionary relationships. This can be at least in part associated with their different substrate specificity. Indeed, group 6 SDIMOs shared the common ancestor with the group 3‐like SDIMOs that include two SDIMO gene clusters named *smoXYB1C1Z* from *Mycobacterium chubuensis* NB44 and *Rhodococcus* sp. ZPP (Zou et al., 2021), which has similar substrate specificity to BCP1 SmoABCD (including various gaseous alkanes and chlorinated hydrocarbons) (Coleman et al., 2012; Martin et al., 2014; Zou et al., 2021). On the other hand, group 5 has a phylogenetic history distinct from the other SDIMO groups probably in association with its high catalytic specificity towards propane. Furthermore, the presence of group 6 SDIMOs only in the genera *Mycobacterium* and *Rhodococcus* and, in some cases, the plasmidic location suggest a more recent acquisition of the group 6 as compared to group 5 SDIMO (that is distributed among different genera and have only chromosomal location) (Vial & Hommais, 2020). The group 6 SDIMO in *Rhodococcus* spp. strains was probably acquired through a horizontal gene transfer (HGT) event from the *M. dioxanotrophicus* lineage to the *R. aetherivorans* lineage. *Rhodococcus* spp. strains frequently carry megaplasmids that act as reservoirs of catabolic functions which greatly contribute to the metabolic diversity and versatility of strains of this genus (Larkin et al., 2005).

The genetic, functional and phylogenetic results from the present work can assist in future strategies of bioremediation by providing information on (i) the molecular targets to detect and quantify in microbial communities applied in co‐metabolic processes of chloroform and trichloroethane and (ii) the genetic functions to introduce in possible microbial cell factories designed to be applied in biodegradation processes (through bioaugmentation in situ or in bioreactors) of soils or waters contaminated by short‐chain *n‐*alkanes and chlorinated hydrocarbons.

## CONCLUSIONS

This study demonstrates the role of the rare SDIMO SmoABCD in the growth and biodegradation of gaseous and liquid *n‐*alkanes and in the biodegradation of chlorinated hydrocarbons in *Rhodococcus aetherivorans* BCP1. The combination of this functional information together with previous genetic and transcriptional analyses indicates that SmoABCD is one of the different alkane hydroxylase systems which are present in *R. aetherivorans* BCP1 and have complementary and integrated roles in the metabolism of alkanes and degradation of chlorinated alkanes. Phylogenetic and comparative approaches indicate that SmoABCD belongs to the group 6 SDIMOs. The latter has been recently acquired by *R. aetherivorans* BCP1 through a horizontal gene transfer (HGT) conferring advantages in terms of alkane degradation kinetics and cellular growth rate. In general, this study provides fundamental information on the functions of a group 6 SDIMO, providing also novel insights into the biological and evolutionary significance of the alkane hydroxylation‐functional redundancy that is at the basis of the metabolic versatility of some biotechnologically relevant bacteria like *Rhodococcus* bacterial strains.

## AUTHOR CONTRIBUTIONS

**Eleonora Ferrari:** Data curation; formal analysis; investigation; visualization; writing – original draft; writing – review and editing. **Giulio Di Benedetto:** Data curation; formal analysis; investigation; writing – original draft. **Andrea Firrincieli:** Software; writing – original draft; writing – review and editing. **Alessandro Presentato:** Investigation; methodology; writing – review and editing. **Dario Frascari:** Formal analysis; methodology; resources; writing – review and editing. **Martina Cappelletti:** Conceptualization; funding acquisition; resources; supervision; visualization; writing – original draft; writing – review and editing.

## FUNDING INFORMATION

This research was supported by internal funding from the University of Bologna (RFO). Open access publishing fees were partly covered by a dedicated funding support provided by the Department of Pharmacy and Biotechnology (FaBit) University of Bologna.

## CONFLICT OF INTEREST STATEMENT

The authors declare no conflict of interest.

## Supporting information


Data S1.

Table S3.

Table S4.

## Data Availability

Raw data used to generate Figures 6 and S7 and the SDIMOs phylogenetic trees in Figures 5A,B and S6 are available under the https://doi.org/10.6084/m9.figshare.24972594. Genome sequence and annotation of *Rhodococcus aetherivorans* BCP1 are available under the NCBI RefSeq assembly GCF_000470885.1.
