# Supplementary material for: Unravelling the role of the group 6 soluble di‐iron monooxygenase (SDIMO) SmoABCD in alkane metabolism and chlorinated alkane degradation
Source: Microb Biotechnol. 2024 Apr 29;17(5):e14453. doi: 10.1111/1751-7915.14453 (PMC11057499; doi:10.1111/1751-7915.14453)
Supplement: Supplementary file 1 — Data S1. [file MBT2-17-e14453-s003.docx]

**Supplementary material**

**_
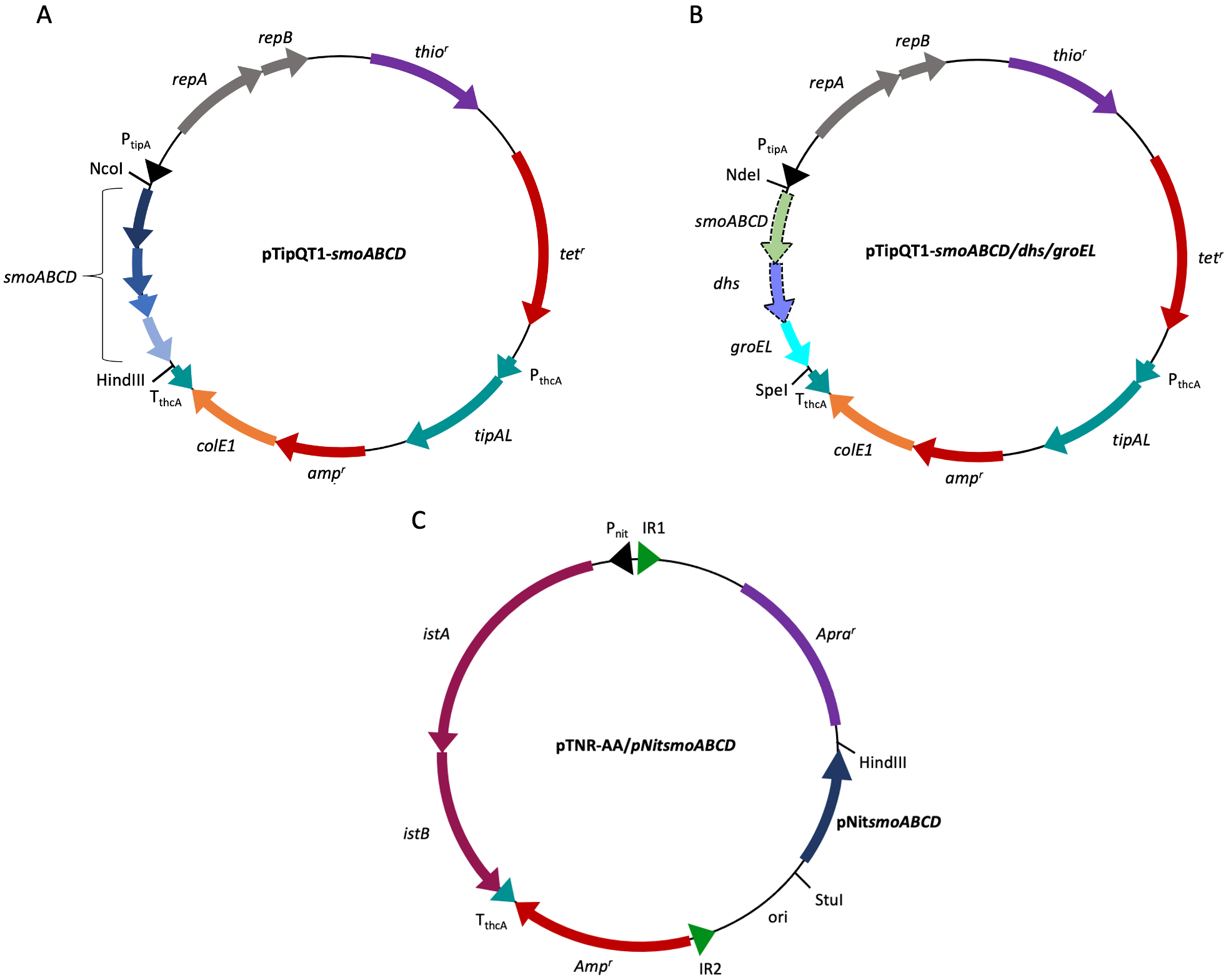
_**

**Figure S1** Schematic representation of the vectors generated in this study, that are the inducible expression vectors pTipQT1-*smoABCD* (A), the pTipQT1-*smoABCD/DHs/groEL* (short-name of *smoABCD/aldDH/alcDH/groEL*) (B), and the non*-*replicating transposon-based vector pTNR-AA/*pNitsmoABCD* (C). P_tipA,_ thiostrepton-inducible promoter; *repAB*, minimum region derived from the cryptic plasmid pRE2895 for autonomous replication of the plasmid in *Rhodococcus*; *thio^r^*, thiostrepton resistance gene; *tet^r^*, tetracycline resistance gene; P_thcA_, thcA promoter that transcribes the *tipAL* gene constitutively; *amp^r^*, ampicillin resistance gene; *colE1*, replication origin for *E. coli*; T_thcA_, thcA transcriptional terminator; P_nit_, constitutive promoter from pNitQT1 (Nakashima & Tamura, 2004); IR1, IR2, inverted-repeat 1 e 2; *apra^r^*, apramycin resistance gene (the sense of transcription is not indicated because it was not defined); ori, origin of replication for *E. coli*; *istA*, *istB*, genes with transposase activity from IS1415.

| 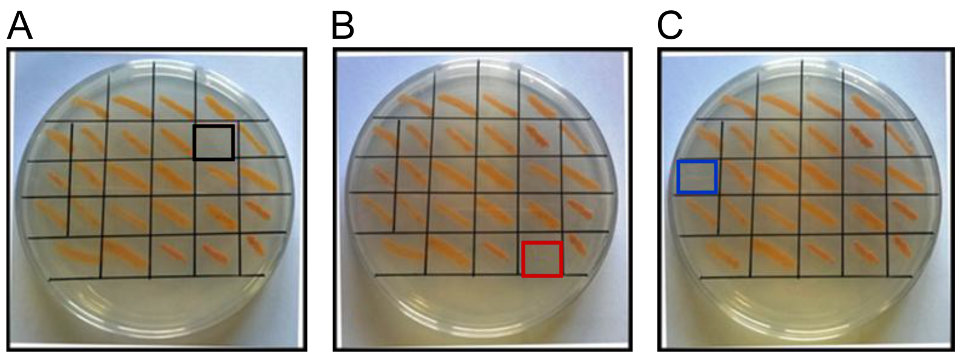  A  B  C |
| --- |

**Figure S2** Examples of *R. aetherivorans* BCP1 transposon mutants streaked on agar plates to screen their ability to grow on *n-*alkanes. BCP1 transposon mutants were analysed for their ability to grow on solid minimal medium (MSM) supplied with thiostrepton (10 µg/ml) (for mutant selection) and (A) glucose (0.5% w/v), (B) hexane (0.1% v/v) (C) or hexadecane (0,1% v/v) as the sole carbon and energy source. Representative mutants are depicted in coloured boxes: a mutant unable to grow on minimal medium supplied with a standard carbon source like glucose is in black (this types of mutants were omitted from further analyses), a mutant defective for the ability to grow on C16 but still able to grow on C6 is in in blue (these types of mutants were not analysed in this work), a mutant that is unable to grown on C6 but retains the ability to grow on C16 is in red (the mutant depicted in this picture is 2.10), a mutant that is able to grow both on standard carbon sources and on both the short- and medium-chain n-alkanes is in green the mutant 2.13 (the mutant depicted in this picture is 2.13 that was used as experimental control to define the effect of *thio^R^* and thiostrepton addition to the output of the experiments)


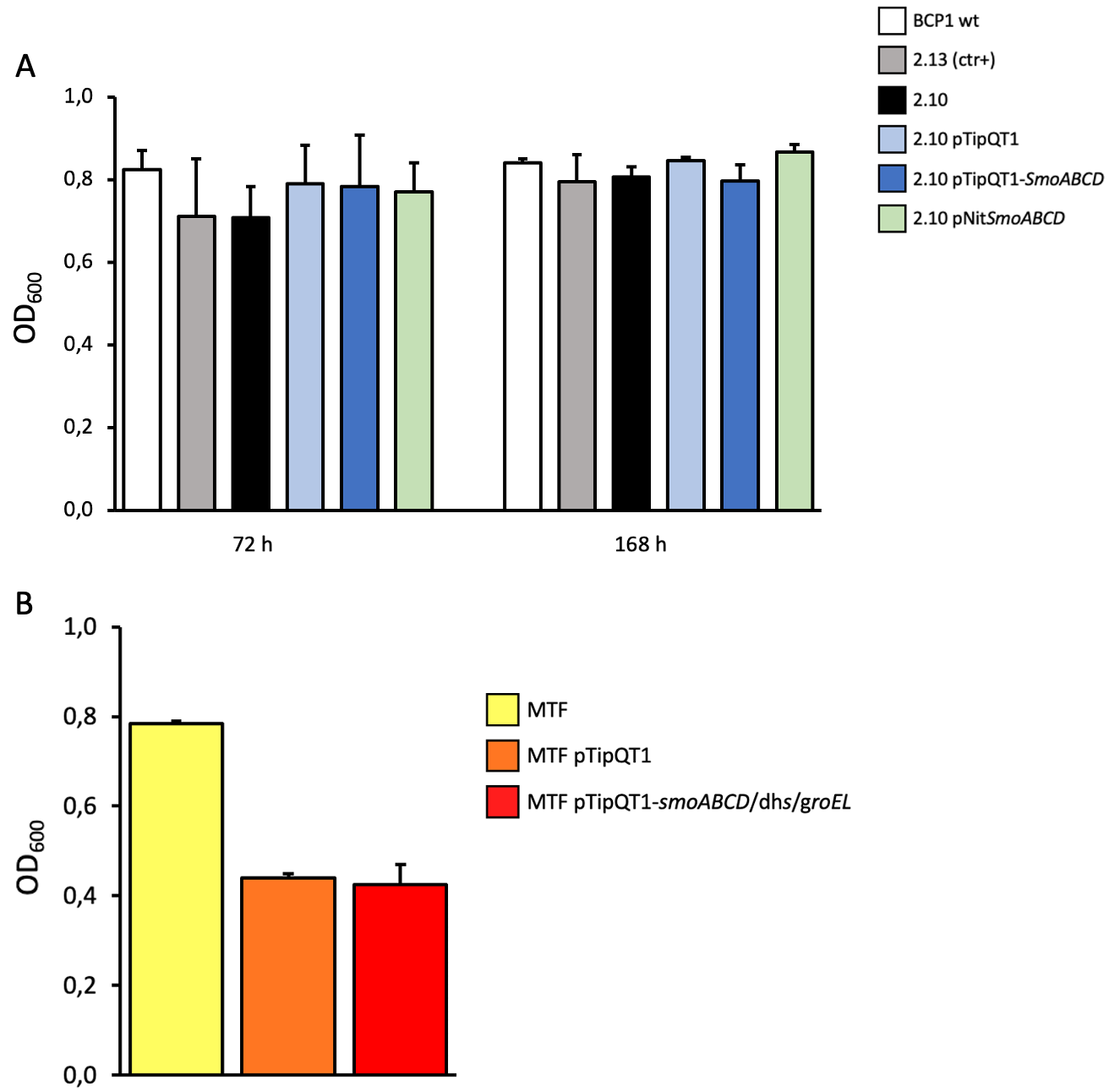


**Figure S3** Growth of *R. aetherivorans* BCP1 WT and mutant strains *Rhodococcus* on glucose. Bacterial growth was measured as optical density (OD) at 600 nm after 72 h and/or 168 h from the initial inoculation in MSM medium supplied with glucose (0.5% w/v) as only carbon and energy source. Results are means ± SD with n=3. Triplicates were performed and each experiment was repeated two times.

| 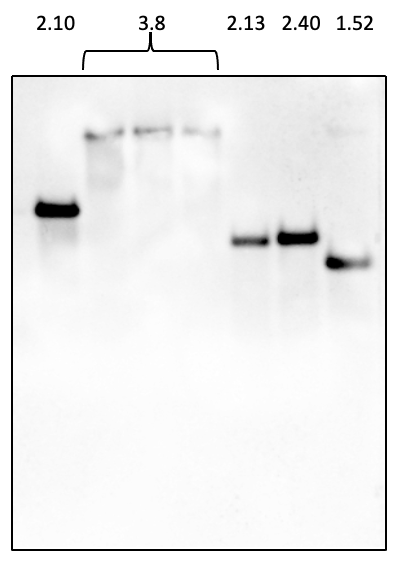 |
| --- |

**Figure S4** Southern blot analysis confirming the single insertion of the pTNR-TA vector in *R*. *aetherivorans* BCP1 mutants. The *thio^R^*-based probe was hybridized with the genomic DNA extracted from different random transposon mutants of *R. aetherivorans* BCP1, i.e., 2.10, 3.8, 2.13, 2.40 and 1.52. Three different genomic DNA preparations of the mutant 3.8 (extracted from three separated cultures) were assayed to test the reproducibility of Southern blot analysis and the absence of aspecific hybridization product.


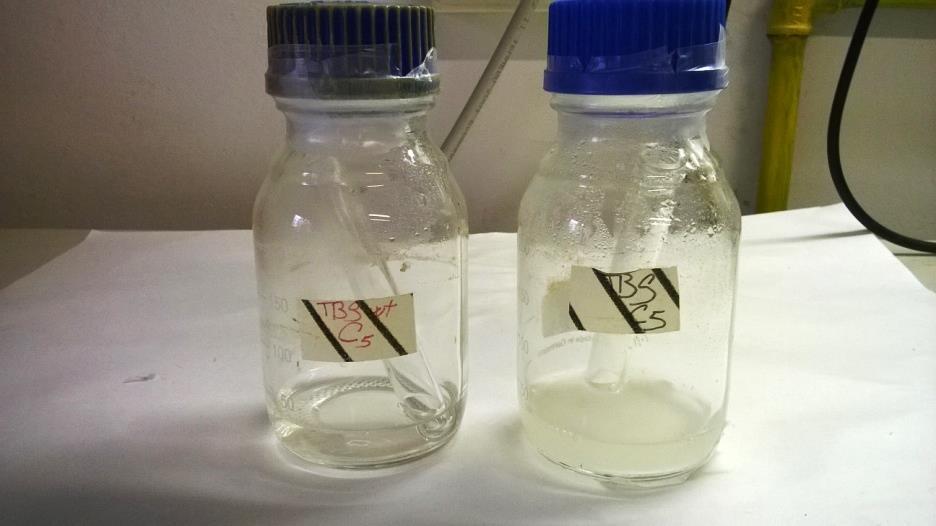

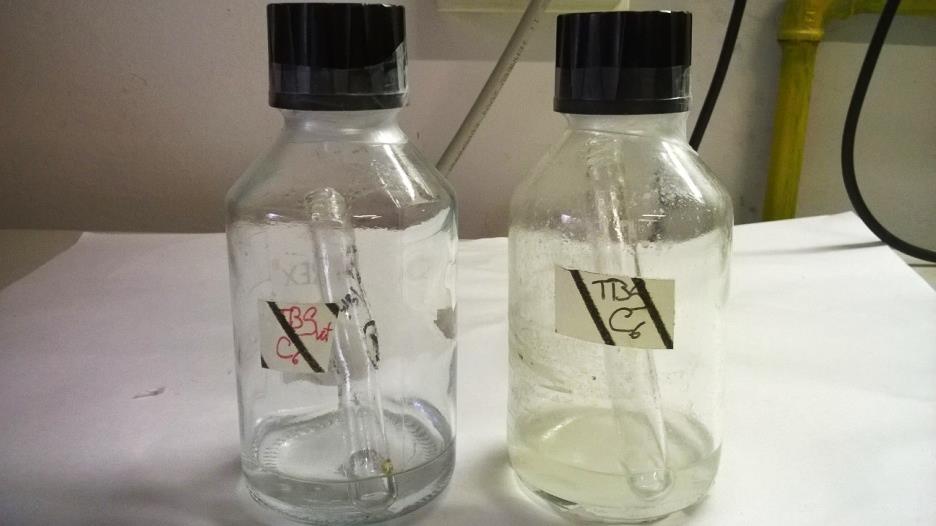


**Figure S5** Pictures of cultures of MTF WT and MTF pTip-*smoABCD/aldDH/alcDH/groEL* grown in the presence of pentane (C5) and hexane (C6) as only carbon and energy source. The alkanes were added inside glass tubes and separated from the liquid medium with the bacterial cells to reduce cellular toxicity due to direct contact and solvent effect.

**Figure S6** Maximum likelihood phylogenetic tree showing the details of the branching structure of all the SDIMO groups (1 – 6) in members of *Mycobacteriaceae* family. Phylogenetic distances in the phylogenetic tree were calculated using the Q.yeast+F+I+G4 substitution model. Ultrafast bootstrap support values > 80 (1000 bootstrap replicates) are indicated as black dots.

(Ald DH)

(Alc DH)

**Figure S7** Organization and conservation of the amino acid products of the *smoABCD* gene cluster and the flanking genes *hisK*, *luxR-like*, *aldDH* and *alcDH* considering all the strains of *Rhodococcus* and *Mycobacterium* strains carrying the sub-group 3 of the group 6 SDIMOs. The *smo* gene clusters are anchored by the *smoA* gene. In grey are coding regions not conserved in the *R. aetherivorans* *smo* gene cluster. Gene-to-gene percent identity is highlighted in grey shade. The Genbank files (*.gbk) obtained from CAGECAT (<https://cagecat.bioinformatics.nl/>) and used to generate the Supplementary figure 7 in clinker (<https://github.com/gamcil/clinker>) are available in Figshare: https://figshare.com/s/65993830925bf949fc95

**Table S1** Bacterial strains and plasmids used in this study

| Strain or plasmid | Relevant genotype or characteristics^a,b^ | Reference |
| --- | --- | --- |
| Bacterial strains | | |
| *Escherichia coli* DH5α | Host strain for cloning and vector construction; *supE44 hsdR17 recA1 endA1 gyrA96 thi-1 relA1* | (Hanahan, 1983) |
| *Rhodococcus aetherivorans* BCP1 | Able to grow on short-chain *n*-alkanes and to co-metabolize chlorinated hydrocarbons | (Frascari et al., 2006) |
| BCP1 2.13 | Transposon insertion mutant of *R. aetherivoran* BCP1; it has no impaired growth phenotype on *n*-alkanes; Thio^R^ | (This work) |
| BCP1 2.10 | Transposon insertion mutant of *R. aetherivorans* BCP1 with *thio^R^* inserted in *smoA* gene; it has limited or absent capacity to grow on short-chain *n*-alkanes C_3_-C_7_; Thio^R^ | (This work) |
| BCP1 2.10 pTipQT1 | *R. aetherivorans* BCP1 2.10 transformed with the empty vector pTipQT1; Thio^R^/Tet^R^  (*Rhodococcus*), Amp^R^ (*E. coli*) | (This work) |
| BCP1 2.10 pTipQT1-*smoABCD* | *R. aetherivorans* BCP1 2.10 transformed with the pTipQT1 vector carrying the *smoABCD* operon under the thiostrepton-inducible promoter pTip; Thio^R^, Tet^R^ (*Rhodococcus*), Amp^R^ (*E. coli*) | (This work) |
| BCP1 2.10 pNit*smoABCD* | *R. aetherivorans* BCP1 2.10 with a single insertion in the genome of the *smoABCD* operon under the consitutive promoter pNit; Thio^R^ and Apra^R^ | (This work) |
| *Rhodococcus erythropolis* MTF | *Rhodococcus* bacterial strain unable to grow on short-chain *n*-alkanes; used for heterologous expression experiment | (Golby et al., 2014) |
| MTF pTipQT1-*smoABCD/aldDH/alcDH/groEL* | *Rhodococcus erythropolis* MTF transformed with pTipQT1 carrying the gene operon *smoABCD* together with the downstream genes encoding an aldehyde DH, an alcohol DH and a GroEL chaperon under the control of the pTip promoter; Thio^R^, Tet^R^ | (This work) |
| *Rhodococcus erythropolis* SQ1 | *Rhodococcus* bacterial strain unable to grow on short-chain *n*-alkanes; tested for heterologous expression experiment | (Quan & Dabbs, 1993) |
| SQ1 pTipQT1- *smoABCD/aldDH/alcDH/groEL* | *Rhodococcus erythropolis* SQ1 transformed with pTip-QT1 carrying the gene cluster *smoABCD/aldDH/alcDH/groEL* under the control of the P_Tip_ promoter; Thio^R^, Tet^R^ | (This work) |
| Plasmids | | |
| pUC18 | Cloning vector; Amp^R^ (*E. coli*) | (Yanish-Perron et al. 1985) |
| pIJ8600 | Shuttle vector *E. coli*-*Streptomyces*; used as origin of *apra^R^* gene; Apra^R^ | (Takano et al. 1995) |
| pTNR-TA | Non*-*replicating transposon-based vector for *Rhodococcus*; Amp^r^ (*E. coli*), Thio^R^ (*Rhodococcus*) | (Sallam et al., 2007) |
| pTNR-AA | Non*-*replicating transposon-based vector for *Rhodococcus;* Amp^R^ (*E. coli*), Apra^R^ (*Rhodococcus*) obtained by replacing (within the BamHI-HindIII RE sites) Thio^R^ with Apra^R^ (from pIJ8600) | (This work) |
| pTNR-AA/*pNitsmoABCD* | Derived from pTNR-AA; it carries the operon *smoABCD* (within StuI- HindIII RE sites) under the control of the constitutive promotor pNit*;* Amp^r^ (*E.coli*), Apra^r^ (*Rhodococcus*) | (This work) |
| pTipQT1 | Shuttle vector *E.coli*-*Rhodococcus*; it has the inducible promoter pTipA and *repAB* genes for replication in *Rhodococcus* (from pRE2895); Amp^r^ (*E. coli*) Tet^R^, Thio^R^ (*Rhodococcus*) | (Nakashima & Tamura, 2004) |
| pTipQT1-*smoABCD* | Derived from pTipQT1; it carries the operon smoABCD (within NdeI-HindIII RE sites) under the control of the thiostrepton inducible promoter pTip; Amp^r^ (*E. coli*), Tet^R^, Thio^R^ (*Rhodococcus*) | (This work) |
| pTipQT1- *smoABCD/aldDH/alcDH/groEL* | Derived from pTip-QT1; it carries the *smoABCD* operon in cluster with the genes *aldDH*, *alcDH* e *groEL* (within NdeI-SpeI RE sites) under the control of under the control of pTip; Amp^r^ (*E. coli*), Tet^R^, Thio^R^ (*Rhodococcus*) | (This work) |
| pNitQT1 | Shuttle vector *E.coli*-*Rhodococcus*; it has the constitutive promoter P_Nit_ and *repAB* genes for replication in *Rhodococcus* (from pRE2895); Amp^R^ (*E. coli*), Tet^R^ (*Rhodococcus*) | (Nakashima & Tamura, 2004) |
| pNitQT1-*smoABCD* | Derived from pNitQT1; it carries the *smoABCD* operon (within NdeI-HindIII RE sites) under the control of pNit; Amp^r^ (*E. coli*), Tet^R^ (*Rhodococcus*) | (This work) |

^a^ RE = restriction enzyme

^b^ Tet^R^=tetracycline resistance, Amp^R^=ampicillin resistance; Tio^R^= tiostrepton resistance, Apra^R^=apramycin resistance

**Table S2** Ability of *R*. *aetherivorans* BCP1 2.10 and 2.13 mutants to grow on short-chain *n-*alkanes metabolic intermediates^a^

| Substrate | Growth (OD_600_)^b^ | |
| --- | --- | --- |
|  | 2.10 | 2.13^c^ |
| 1-Propanol | 0.96±0.021 | 0.82±0.012 |
| 1-Butanol | 0.85±0.185 | 0.98±0.004 |
| 1-Hexanol | 0.42±0.007 | 0.56±0.014 |

^a^ Mean values ± SD

^b^ The growth was measured as OD_600_ after 72 hours of incubation

^c^ The mutant 2.13 was used a control as it carries *thio^R^* but it is not impaired in short-chain *n-*alkanes growth
